# Endotoxin Induced Chorioamnionitis Prevents Intestinal Development during Gestation in Fetal Sheep

**DOI:** 10.1371/journal.pone.0005837

**Published:** 2009-06-08

**Authors:** Tim G. A. M. Wolfs, Wim A. Buurman, Bea Zoer, Rob M. J. Moonen, Joep P. M. Derikx, Geertje Thuijls, Eduardo Villamor, Markus Gantert, Yves Garnier, Luc J. I. Zimmermann, Boris W. Kramer

**Affiliations:** 1 Department of Surgery, Nutrition and Toxicology Research Institute Maastricht (NUTRIM), Maastricht University Medical Centre (MUMC), Maastricht, the Netherlands; 2 Department of Pediatrics, Maastricht University Medical Center (MUMC), School of Oncology and Developmental Biology (GROW), Maastricht, the Netherlands; 3 Department of Obstetrics & Gynecology, University of Cologne, Cologne, Germany; University of Cambridge, United Kingdom

## Abstract

Chorioamnionitis is the most significant source of prenatal inflammation and preterm delivery. Prematurity and prenatal inflammation are associated with compromised postnatal developmental outcomes, of the intestinal immune defence, gut barrier function and the vascular system. We developed a sheep model to study how the antenatal development of the gut was affected by gestation and/or by endotoxin induced chorioamnionitis.

Chorioamnionitis was induced at different gestational ages (GA). Animals were sacrificed at low GA after 2d or 14d exposure to chorioamnionitis. Long term effects of 30d exposure to chorioamnionitis were studied in near term animals after induction of chorioamnionitis. The cellular distribution of tight junction protein ZO-1 was shown to be underdeveloped at low GA whereas endotoxin induced chorioamnionitis prevented the maturation of tight junctions during later gestation. Endotoxin induced chorioamnionitis did not induce an early (2d) inflammatory response in the gut in preterm animals. However, 14d after endotoxin administration preterm animals had increased numbers of T-lymphocytes, myeloperoxidase-positive cells and gammadelta T-cells which lasted till 30d after induction of chorioamnionitis in then near term animals. At early GA, low intestinal TLR-4 and MD-2 mRNA levels were detected which were further down regulated during endotoxin-induced chorioamnionitis. Predisposition to organ injury by ischemia was assessed by the vascular function of third-generation mesenteric arteries. Endotoxin-exposed animals of low GA had increased contractile response to the thromboxane A2 mimetic U46619 and reduced endothelium-dependent relaxation in responses to acetylcholine. The administration of a nitric oxide (NO) donor completely restored endothelial dysfunction suggesting reduced NO bioavailability which was not due to low expression of endothelial nitric oxide synthase.

Our results indicate that the distribution of the tight junctional protein ZO-1, the immune defence and vascular function are immature at low GA and are further compromised by endotoxin-induced chorioamnionitis. This study suggests that both prematurity and inflammation in utero disturb fetal gut development, potentially predisposing to postnatal intestinal pathology.

## Introduction

Chorioamnionitis is a bacterial infection of the amniotic fluid, placenta and membranes that is very frequently diagnosed after preterm birth [1]. The disorder is usually clinically silent which makes estimates about the onset of infection during gestation difficult [2]. Chorioamnionitis results from the fetal exposure to bacteria and bacterial toxins in the contaminated amniotic fluid. Animal studies showed that fetal aspiration of contaminated amniotic fluid induces lung inflammation, resulting in profound changes of the pulmonary homeostasis as indicated by increases in surfactant lipids and transient injury to the microvasculature and alveolar septa [3]–[6]. These alterations in lung development are associated with increased risk of bronchopulmonary dysplasia [7].

Swallowing of the bacterial contaminated amniotic fluid is likely to result in antenatal exposure of the premature intestine to bacteria and their toxins in utero. Whether this exposure results in inflammatory and or developmentary changes of the gut of preterm babies is subject of the current study. We hypothesized that prematurity and inflammation, either alone or combined, disturb maturation of the gut barrier, the innate immune defense and vascular function thereby potentially increasing the risk to postnatal intestinal pathologic conditions. To test our hypothesis, chorioamnionitis was induced by intraamniotic endotoxin injection at low gestational age (GA) and animals were delivered either preterm or near term. In this model, the distribution of the tight junctional protein zonula occludens protein 1 (ZO-1) was determined since no data exists on its expression during gestation. Besides the TLR4 and MD-2 mRNA expression, also the distribution of either myeloperoxidase (MPO) expressing cells (polymorphonuclear leukocytes (PMN) as marker for early inflammation) or T-lymphocytes and gammadelta T-cells (cells known to be crucial in monitoring and maintaining integrity of epithelial tissues; late inflammation) were assessed. Finally, the contraction or relaxation of third-generation mesenteric arteries was studied. Relaxation was studied in the absence and presence of a NO donor and concomitantly the expression of endothelial nitric oxide synthase (eNOS) was assessed.

In this study, we show that a low gestational age and endotoxin induced chorioamnionitis both prevent maturation of the intestinal barrier function, the innate immune defense and vascular function.

## Materials and Methods

### Animals

**Figure 1:**
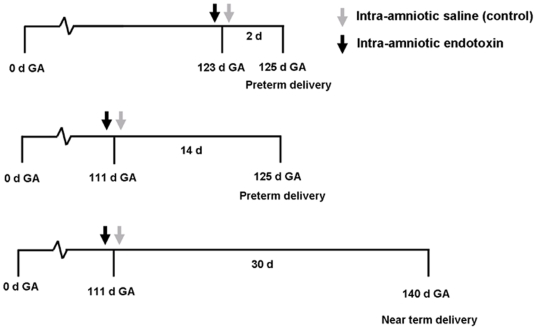
Experimental design. Gestational development and effects of chorioamnionitis were studied at two different gestational ages. At 125d GA, fetuses were comparable to 27 weeks of human gestation. Lambs were almost near term at 140d GA since term gestation is 147d. Chorioamnionitis was induced by a single injection of endotoxin under ultrasound guidance at 111d GA and at 123d GA. Animals were delivered at 125d GA and animals of the control group underwent the same procedure with an injection of saline. Animals of 140d GA had a separate control group to assess gestational changes.

This study was performed according to the guidelines of the Animal Care Committee of the University of Maastricht, which approved the protocol. The study was conducted after approval and in compliance with the guidelines of the ethical committee. Time-mated Texel ewes with singleton fetuses were randomly assigned to groups of four or five animals, to receive a single dose of 10 mg endotoxin (*Escherichia coli* 055:B5; Sigma Chemical, St. Louis, MO) resuspended in saline or the equivalent volume of saline for control animals by ultrasound guided intraamniotic injections (Figure 1). Chorioamnionitis was induced at 110–111d GA and at 123d GA. Animals were sacrificed at low GA of 125d GA after 2d or 14d exposure to chorioamnionitis. The low GA of 125d GA is comparable with a human GA of approximately 27 weeks. Long term effects of 30d exposure to endotoxin were studied in near term animals at 140d GA (term gestation being 147d GA) after induction of chorioamnionitis at 110–111d (Figure 1).

All animals were delivered by caesarean section. Lambs received a lethal i.v. injection of phenobarbiturate. The stomach content was collected for analysis. Samples of the small intestine were collected for snap freezing and fixation. Third generation mesenteric arteries of the small intestine were prepared for analysis of vascular reactivity.

### Antibodies and reagents

Intestinal tissue was analysed with the following antibodies: monoclonal antibody against endothelial nitric oxide synthase (eNOS) (BD Transduction laboratories, San Jose, CA); rabbit antibodies against human MPO and CD3 Dakocytomation (Glostrup, Denmark) or Zonula Occludens protein 1 (ZO-1) (Invitrogen, San Francisco, CA); monoclonal antibody IL-A29 for the detection of gammadelta T-cells (VMRD, Pullman, WA). Secondary antibodies, biotin conjugated rabbit anti-mouse and Texas red conjugated goat anti-rabbit were purchased from Dakocytomation and peroxidase conjugated goat anti-rabbit from Jackson (West Grove, PA).

### Immunohistochemistry

Small intestinal biopsies, fixed overnight in 4% formaldehyde, were embedded in paraffin and sectioned at 3 µm. For staining of CD-3 and eNOS expressing cells, slides were heated in 10 mM Na-citrate (pH 6.0) for 20 min. Endogenous peroxidase activity was blocked with 0.3% H_2_O_2_ in methanol and subsequently, slides were blocked with either normal goat serum (eNOS and MPO) or bovine serum albumin (CD-3) for 30 min at room temperature. Slides were incubated with the primary antibody of interest for 1 h at RT (CD-3 and MPO) or overnight at 4°C (eNOS). After washing, sections were incubated with the appropriate secondary conjugated antibody. Binding of the eNOS antibody was demonstrated by the streptavidin-biotin system (Dakocytomation) and binding of antibodies against CD-3 and MPO was detected using a peroxidase conjugated secondary antibody. Positive staining was visualized by applying 3-amino-9-ethylcarbazole (AEC, Sigma); nuclei were counterstained with haematoxylin. The number of cells exhibiting immunostaining for MPO and CD-3 were counted per high power field (200x). Figures were expressed as number of MPO or CD-3 expressing cells per high power field.

Frozen tissue sections were first fixed in filtered, ice-cold acetone and next in 4% paraformaldehyde. Endogenous peroxidase was blocked with 0.3% H_2_O_2_ in methanol. After blocking with 10% normal goat serum, sections were incubated for 2 h with a mab against gamma delta T-cells. After washing, an appropriate biotin conjugated secondary antibody was used. Binding of the primary antibody was demonstrated by the streptavidin-biotin system (Dakocytomation) and visualized by AEC. Nuclei were counterstained with haematoxylin. All slides were photographed by a Nikon eclipse E800 microscope with a Nikon digital camera DXM1200F and were analysed by the computer program, Lucia G color image analysis.

### Immunofluoresence

Tight junction distribution was examined by immunofluorescent staining of frozen sections (3 µm) with an antibody to Zonula Occludens protein 1 (ZO-1). Ileum sections were fixed with 4% paraformaldehyde. After blockage of non-specific binding sites with 10% goat serum, slides were incubated with anti-ZO-1 for 1 h. Thereafter, the sections were incubated 45 min with Texas red conjugated goat anti-rabbit antibody, followed by 2 min incubation with 4′,6-diamino-2-phenyl indole (DAPI), dehydrated in ascending ethanol series and mounted in Fluorescence Mounting Solution (Dakocytomation). The distribution of ZO-1 was recorded at a magnification of 200x using the Metasystems Image Pro System (black and white charge-couple device camera; Metasystems, Sandhausen, Germany) mounted on a Leica DM-RE fluorescence microscope (Leica, Wetzler, Germany). All images were taken at equal time-exposures after being normalized to negative control sections without primary antibody, to exclude non-specific binding of the secondary antibody or autofluorescence. At least 25 microscopic fields for each tissue section were studied.

### Evaluation of mRNA levels by RT-PCR

For RT-PCR total RNA was extracted from ileal tissues using the SV Total RNA isolation system (Promega, Madison, WI). Next, isolated total RNA was treated with RQ1 RNase-Free DNase (Promega) and reverse transcribed using oligo (dT) primer and Moloney murine leukemia virus reverse transcriptase (Life technologies, Paisley, United Kingdom) according to the supplier's recommendations. cDNA samples were standardized based on the content of GAPDH cDNA as housekeeping gene. GAPDH cDNA was evaluated by performance of a GAPDH PCR on multiple dilutions of each cDNA sample. The amount of amplified product was estimated by densitometry of ethidiumbromide stained 1.2% agarose gels using a CCD camera and Imagemaster VDS software (Amersham, Uppsala, Sweden). Primer sequences for GAPDH were:5′-CGT CTT CAC CAC CAT GGA GA-3′ (sense primer) and 5′-CGG CCA TCA CGC CAC AGT TT–3′ (antisense primer); primers used for the amplification of TLR4 mRNA: 5′- CCT CTC CAC CTT GAT ACT GAC G -3′ (sense primer) and 5′- GGT ACC TGG TTC AAT AAA GCC T-3′ (antisense primer) and primers designed for amplification of MD-2 mRNA: 5′- CCT GTT TTC TTC CAT ATT TAC TG (sense primer) and 5′- AAT AAC TTC TTT GCG CTT TGG–3′. PCR reactions with GAPDH, TLR4 or MD-2 specific primers were performed using appropriate dilutions of the cDNA. PCR reactions were performed in a total volume of 25 µl in PCR buffer (Perkin Elmer/Cetus, Emeryville, CA) in the presence of 0.2 mM dNTP (Amersham), 1.0 µM of each primer, 0.3 mM MgCl_2_ and 0.5 U Taq polymerase (Perkin Elmer). PCR conditions for each primer couple were as follows: GAPDH: 95°C for 30 sec, 55°C for 30 sec and 72°C for 30 sec during 21 cycles; TLR4: 95°C for 30 sec, 54°C for 30 sec and 72°C for 30 sec during 40 cycles; MD-2: 95°C for 30 sec, 52°C for 30 sec and 72°C for 30 sec during 33 cycles. Levels of TLR4 and MD-2 RNA expression were evaluated by densitometric image analysis as described above. Relative TLR4 and MD-2 mRNA levels were calculated by comparison of band intensities of the RT-PCR products to standard curves prepared by PCR amplifications on dilution series of a highly concentrated ileal ovine cDNA.

### Arterial reactivity

Since ischemia as a consequence of vascular compromise is one of the frequently proposed risk factors for impaired intestinal homeostasis, responsiveness to contractile and endothelium-dependent and -independent relaxant agonists was assessed in isolated third-generation mesenteric arteries. Artery rings were mounted in a wire myograph, bathed in Krebs solution (gassed with 95% air/5% CO2, pH 7.4), and normalized to a resting pretension corresponding to an intraluminal pressure of 40 mm Hg (125d GA) or 50 mm Hg (140d GA). The contractions induced by KCl (62.5 mM), the thromboxane A2 mimetic U46619 (10^−10^–10^−7^M) and the adrenergic receptor agonist norepinephrine (NE; 10^−10^–10^−4^M) was assessed. In addition, the endothelium-dependent relaxation (evoked by acetylcholine, Ach;10^−9^–10^−4^M) [8] and endothelium–independent relaxation evoked by the NO donor sodium nitroprusside (SNP; 10^−9^–10^−4^M) were analyzed. For the study of the relaxant responses, vessels were pre-contracted with U46619 (10^−7^M) as previously described [9].

### Statistical analysis

The number of cells exhibiting immunostaining for MPO and CD-3 were counted per high power field. A Mann-Whitney *U-*test was used for between-group comparisons. Vascular contractile and relaxation responses are represented as mean±SEM and the 2-way unpaired t-test was used to test for significant differences between saline and endotoxin treated groups. TLR4 and MD-2 mRNA levels were represented as mean±SEM and differences between preterm and term lambs or saline and endotoxin injected animals were compared using an unpaired t-test. Statistical calculations were made using SPSS 15.0 for Windows (SPSS, Chicago, IL) and differences were considered statistically significant at p<0.05.

## Results

### Tight junctions

**Figure 2:**
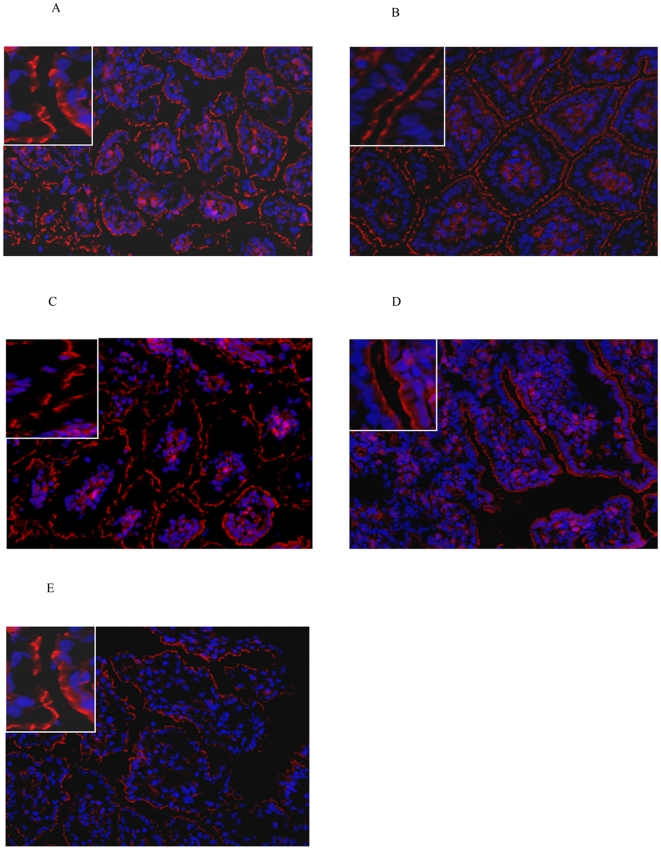
Immunolocalisation of ZO-1 (red) in the fetal intestine. At 125d GA, a fragmented staining pattern for ZO-1 was seen in control animals (A) or lambs exposed to endotoxin for 2 (B) or 14d (C). At 140d GA, a normal ZO-1 distribution was detected in control animals (D) that became disrupted upon endotoxin exposure for 30d (E). Magnification 200x. For inset, 1000x magnification was used.

Tight junctions have an important role in maintaining the barrier integrity by connecting enterocytes [10], [11]. In order to analyse the maturation and the influence of endotoxin in the fetal gut on this essential component of the gut barrier, the distribution of the zonula occludens protein 1 (ZO-1) was determined. ZO-1 staining was fragmented in premature saline treated control animals of 125d GA (Figure 2A). Interestingly, endotoxin exposure for 2d did not change the pattern (Figure 2B). Fetuses exposed to endotoxin for 14d showed further fragmented tight junctional distribution at 125d GA (Figure 2C). In contrast, in near term saline injected animals of 140d GA, ZO-1 proteins formed a continuous ring, which is indicative for a normal tight junctional distribution (Figure 2D). Interestingly, in these near term lambs of 140d GA, exposure to endotoxin for 30d resulted in partial loss of the ZO-1 protein in comparison to the age-matched control animals with saline injection (Figure 2E).

### Inflammatory parameters

**Figure 3:**
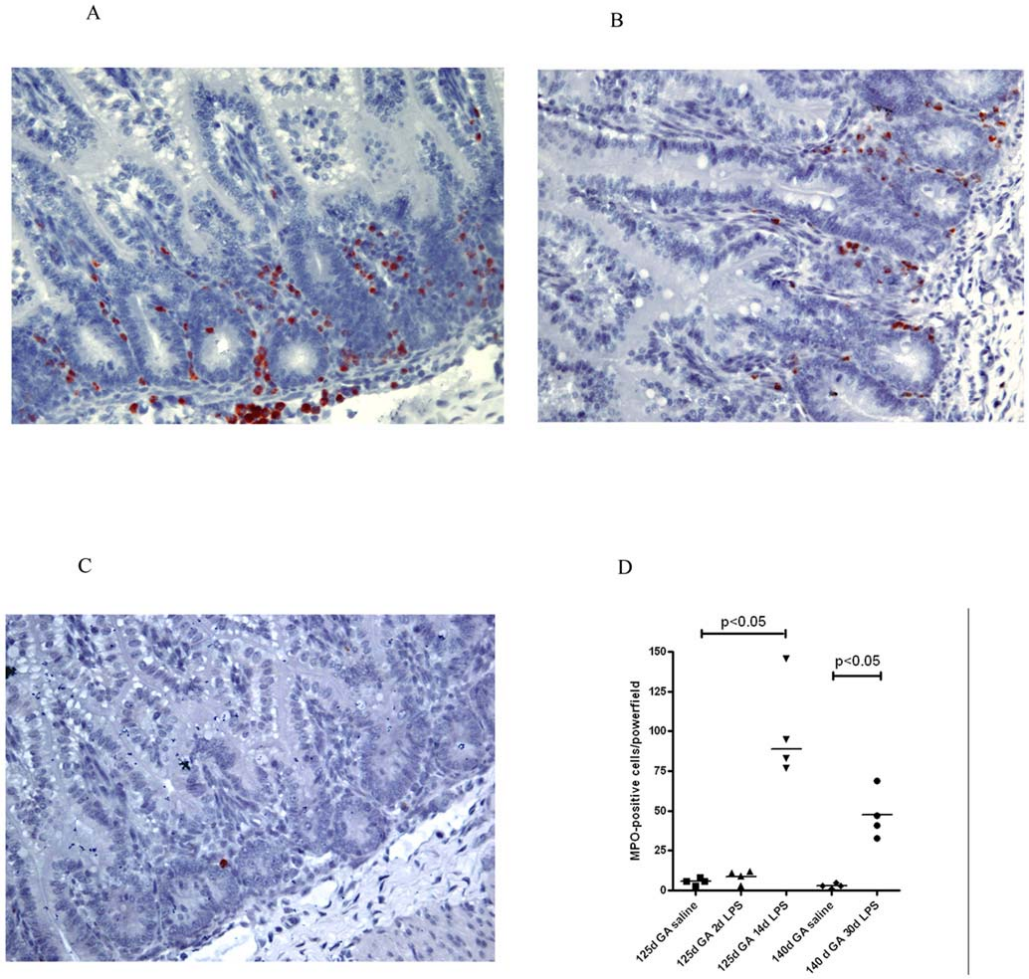
At 14 and 30d after intraamniotic endotoxin injection, massive influx of PMNs was detected (A+B). At 140d GA, some MPO positive cells were detected in control sections (C). For each experimental group mean cell counts of MPO positive cells are depicted per high-power field (D).

In preterm control animals of 125d GA, hardly any MPO expressing cells were detected (data not shown). At this GA, endotoxin exposure for 2d did not result in a significant increase of infiltrating intestinal MPO positive cells (data not shown). Interestingly, at 14 days post endotoxin treatment, large numbers of MPO positive cells were present in the lamina propria of the immature intestine (Figure 3A). Remarkably, 30d post endotoxin treatment, the number of MPO expressing cells reactivity was still significantly enhanced in the near term fetus (Figure 3B) when compared to saline treated control animals of this GA (Figure 3C). For each individual animal, the number of infiltrating MPO expressing cells was counted per high-powerfield and displayed in a scatter plot (Figure 3D).

**Figure 4:**
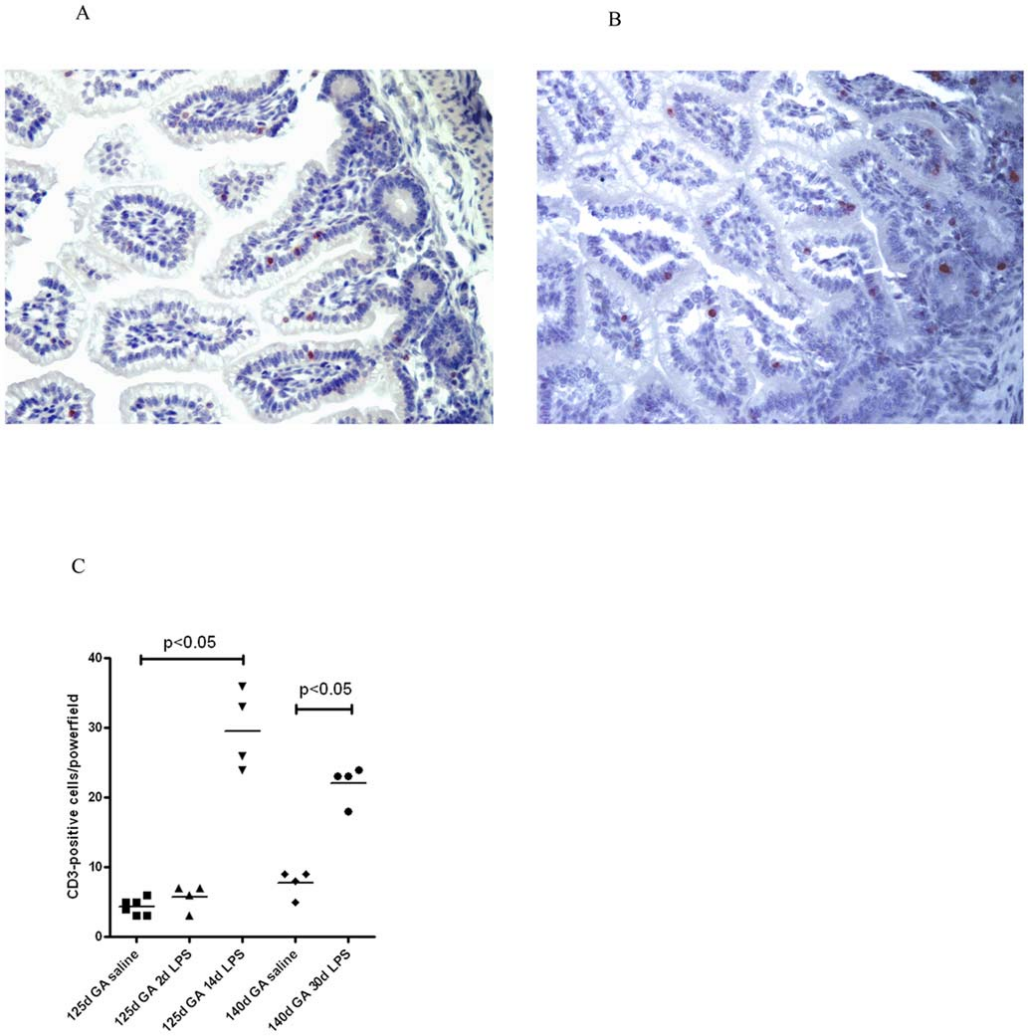
Intraamniotic endotoxin results in increased ileal T-cell density. Significant increase of CD-3 immunoreactivity was seen at 14 and 30d after endotoxin exposure (A+B). For each experimental group mean cell counts of CD-3 positive cells are depicted per high-power field (C).

The recruitment of CD-3 positive T-cells into the fetal gut paralleled that of PMN: in preterm lambs exposed to endotoxin for 2d, no significant increase of CD-3 expressing T-cells in the lamina propria was observed when compared with time matched control tissues of 125d GA (data not shown). In contrast, increased numbers of CD-3 expressing cells were seen at 14d and 30d post endotoxin exposure in both preterm and near term animals of 125 and 140d GA respectively, with the highest density in the 14d group (Figure 4A+B). For each individual section, the number of CD-3 positive cells was quantified per high-powerfield and data are depicted in a scatter plot (Figure 4C).

**Figure 5:**
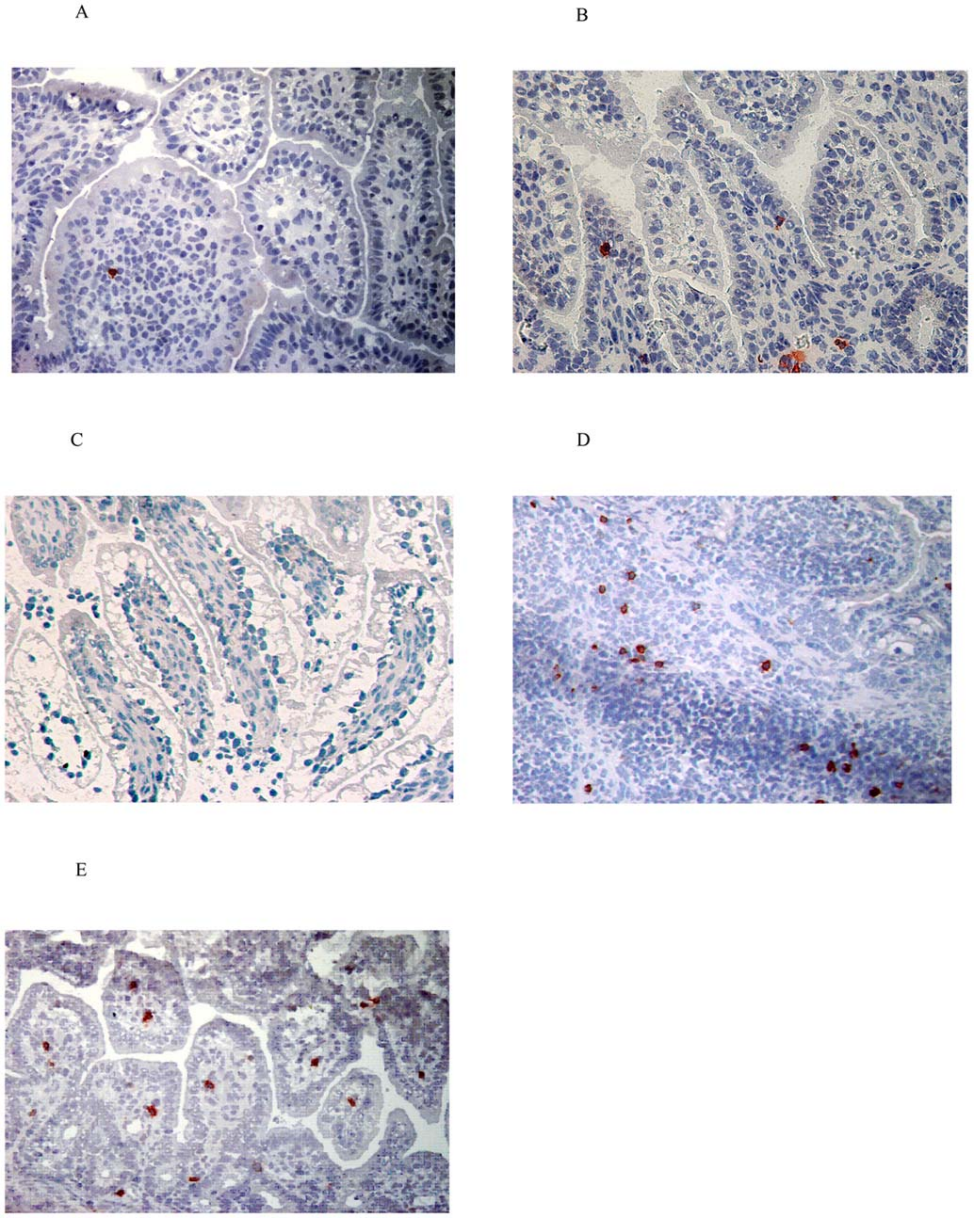
Endotoxin induced chorioamnionitis results in migration of gammadelta T-cells to sites of mucosal damage at late GA. After 2d or 14d of endotoxin exposure, hardly any gammadelta T-cells were detected in the upper mucosa of the preterm intestine (A+B). At 140d GA, gammadelta T-cells were not detected in the lamina propria whereas lymphoid follicles in the basal mucosa became populated with gamma delta T-cells (C+D). 30d after endotoxin injection, increased numbers of gammadelta T-cells were found within areas of mucosal damage (E).

Next, the distribution of gammadelta T-cells was evaluated, cells known to be crucial in monitoring and maintaining integrity of epithelial tissues [12]. In saline treated preterm animals of 125d GA, hardly any gammadelta T-cells were present in the lamina propria or lower mucosa (data not shown). Accordingly, in these preterm lambs, migration of gammadelta T-cells from the lower to the upper mucosa occurred sporadically after 2 or 14d post endotoxin treatment (Figure 5A+B). In saline injected animals of 140d GA, gammadelta T-cells were also undetectable in the lamina propria (Figure 5C). However, in these near term lambs, the lymphoid follicles in the lower mucosa became populated with gammadelta T-cells (Figure 5D). Interestingly, 30 d of intraamniotic endotoxin exposure in near term animals resulted in migration of gammadelta T-cells from the lower to the upper mucosa in close proximity to areas of damaged intestinal epithelial cells (Figure 5E).

### TLR4 and MD-2 expression

**Figure 6:**
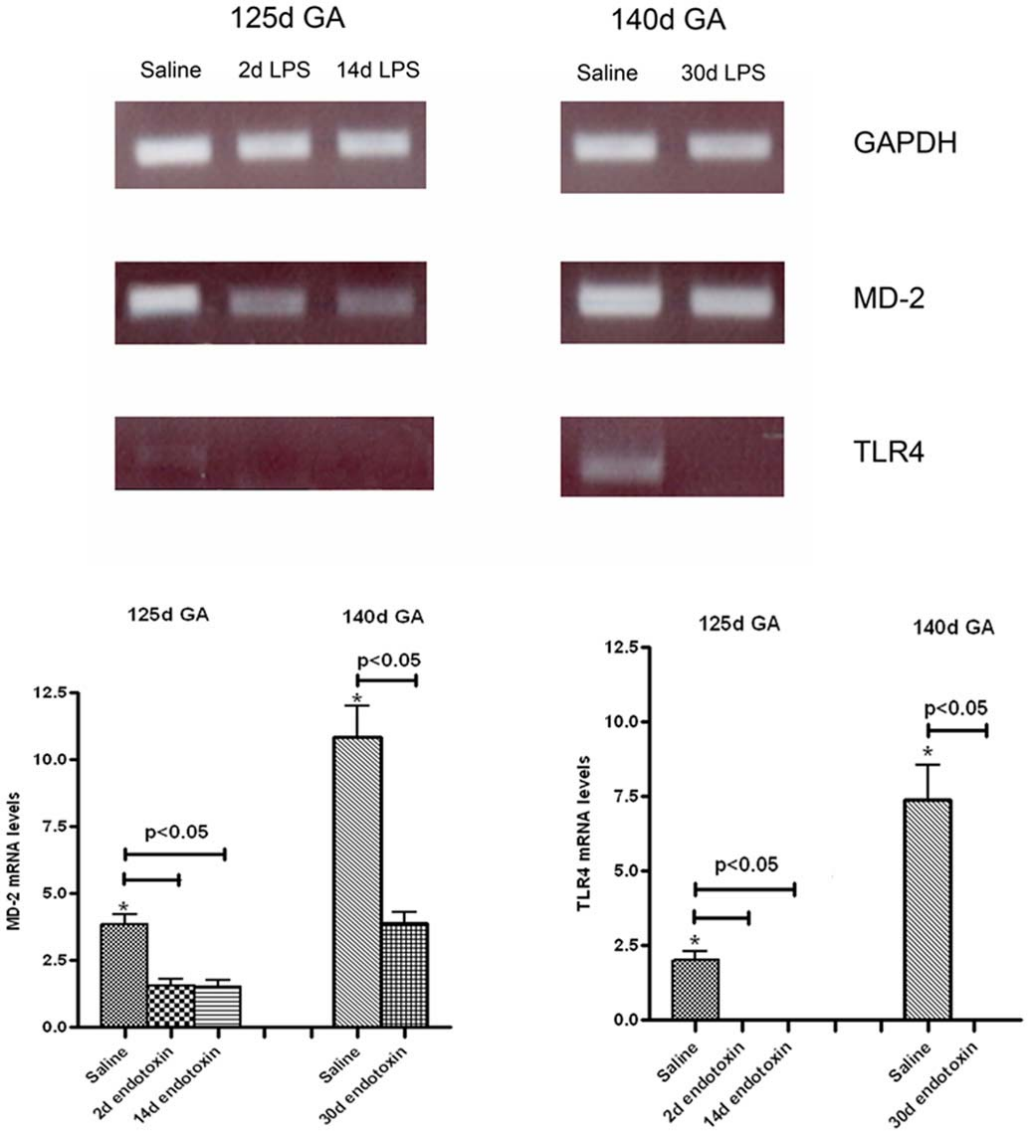
Ileal TLR4 and MD-2 mRNA was evaluated by ovine specific RT-PCR amplification of cDNA samples from healthy control lambs and lambs subjected to endotoxin induced chorioamnionitis. Representative DNA fragments are shown for each group (n = 4). cDNAs were standardized for GAPDH content. Quantitative data were obtained by densitometric evaluation of RT-PCR products which were compared to a standard curve obtained by amplification of a serial dilution of highly concentrated cDNA. At 125d GA, TLR4 and MD-2 mRNA levels were significantly (*) lower compared to animals at 140d GA. TLR4 and MD-2 mRNA expression was significantly reduced following intraamniotic endotoxin injection for 2, 14 and 30d.

The molecular complex responsible for the recognition and signalling of endotoxin is formed by TLR4 and its indispensable cofactor MD-2 [13], [14]. In preterm lambs of 125d, weak constitutive TLR4 and MD-2 mRNA expression was detected and this expression even decreased within 2d following intraamniotic endotoxin treatment (Figure 6). Also intraamniotic exposure to endotoxin for 14d in preterms resulted in a downregulation of the TLR4 and MD-2 mRNA levels (Figure 6). In saline treated near term sheep of 140d GA, TLR4 and MD-2 expression was significantly increased when compared with preterm controls of 125d GA. However, intraamniotic endotoxin exposure for 30d also resulted in a strong reduction of the TLR4 and MD-2 expression in near term lambs (Figure 6).

### Vascular contraction and relaxation responses

**Figure 7:**
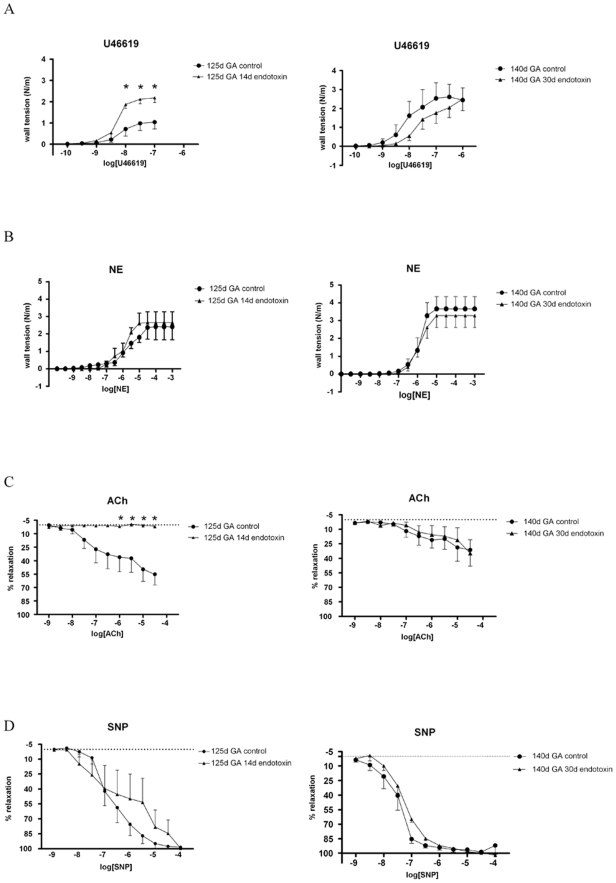
Thromboxane A2 mimic U46619 induced contractions of jejunal arteries increased with gestational age and were significantly (*) (P<0.05) augmented after intra-amniotic endotoxin injection in the 125d (P<0.05) but not in the 140d GA animals (A). Concentration-dependent contractile effects of norepinephrine tend to increase (p>0.05) during gestation but remained unaltered during chorioamnionitis (B). Concentration-dependent relaxation of mesenteric arteries induced by acetylcholine was unaltered during gestation and was significantly (*) inhibited at 14d but not after 30d of endotoxin exposure (C). The relaxation response curve for sodium nitroprusside remained identical during gestation or chorioamnionitis (D).

Contractions of third-generation mesenteric arteries, evoked by the thromboxane receptor agonist U46619 increased with gestational age (Figure 7A). After endotoxin-induced chorioamnionitis, the potency and the contractile efficacy of U46619 was significantly increased at early (125d) but not at late GA (140d) (Figure 7A). In contrast, the contractions induced by KCl (data not shown) and NE (Figure 7B) were not statistically different, both in control animals at different gestational ages and in lambs exposed to chorioamnionitis.

Either ACh (Figure 7C) or SNP (Figure 7D) relaxed U46619-contracted fetal mesenteric arteries in a concentration-dependent manner and these relaxations did not change with gestational age. Interestingly, ACh-evoked relaxation was almost abolished in the 125d fetuses exposed to endotoxin (Figure 7C). However this effect of endotoxin on ACh-induced relaxation was not further observed at 140d GA (Figure 7C).

Interestingly, the presence of SNP completely reversed the impaired relaxation of premature animals exposed to endotoxin (Figure 7D). These findings suggest a vascular impairment during chorioamnionitis at low GA on account of reduced release of NO by endothelial cells.

### eNOS expression

**Figure 8:**
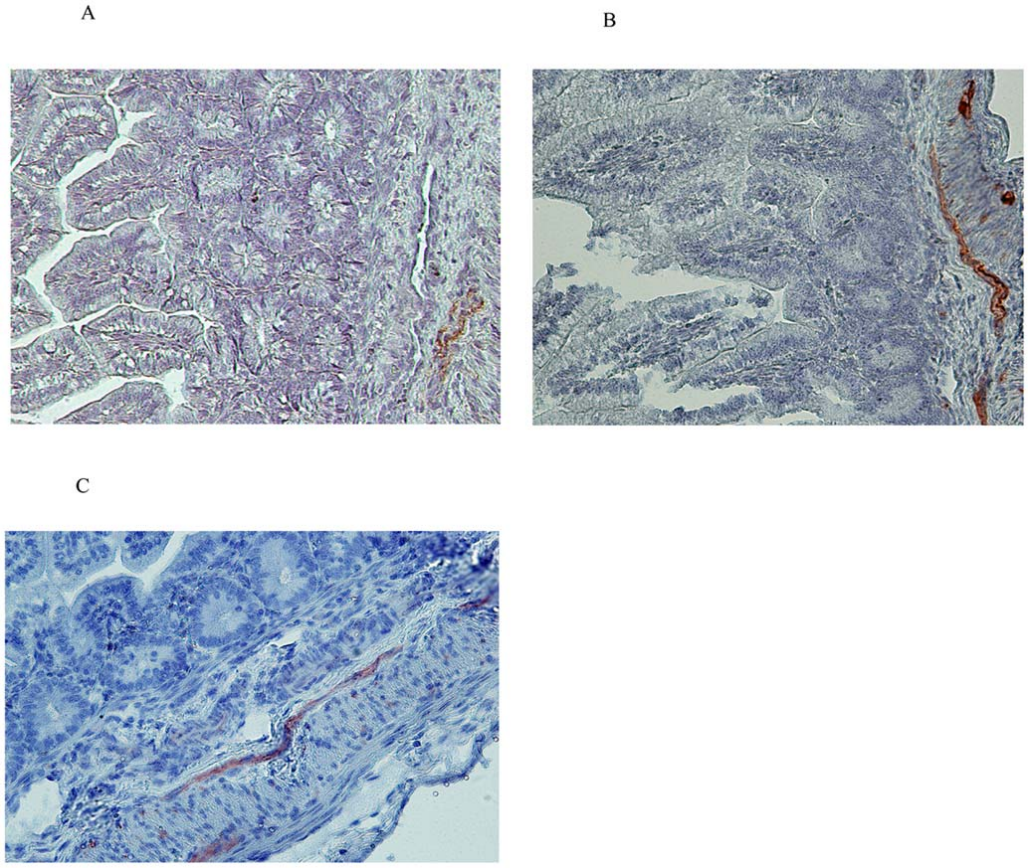
Intraamniotic endotoxin induces eNOS expression. Weak eNOS staining was seen at 2d post endotoxin treatment (A) while expression increased in animals exposed to endotoxin for 14 or days 30d (B-C).

Since the findings described above point at endothelial dysfunction due to impaired synthesis of NO, the distribution of endothelial nitric oxide synthase (eNOS) was studied in mesenteric arteries. Constitutive eNOS expression was absent in preterm and near term control tissues of 125 and 140d GA respectively (data not shown). However, 2 days after intraamniotic endotoxin injection in preterm animals, eNOS was weakly expressed by submucosal vascular endothelia (Figure 8A). At this GA, endothelial staining further enhanced at 14 days after endotoxin exposure (Figure 8B). eNOS immunoreactivity was also present in the vascular endothelium of the submucosa in near term animals that were exposed to endotoxin for 30d (Figure 8C).

## Discussion

This study shows for the first time the effects of gestation either alone or combined with antenatal exposure to bacterial toxins on the development of the fetal gut. A sheep model was used that closely resembles the human situation of preterm babies that are in utero exposed to chorioamnionitis by bacterial toxins resulting in adverse outcomes [15]. Administration of endotoxin to the amniotic fluid resulted in increased concentrations of endotoxin activity in the stomach fluid of animals exposed for 2d to endotoxin (data not shown), indicating passage of endotoxin to the fetal gut. This experimental set up allowed us to investigate whether prematurity and inflammation, either alone or combined, influence the development of the intestine in utero.

To our knowledge, this is the first report showing that the cellular distribution of tight junction protein ZO-1 was underdeveloped at 125d GA in sheep. Although ZO-1 is not the only tight junction protein responsible for paracellular barrier sealing, it plays a central role in maintenance of paracellular intestinal barrier integrity by connection of the intercellular tight junction proteins (claudins and occludin) with the cell cytoskeleton.

The presence of fragmented tight junctions in the gut of preterms (125d GA) is inherent to immaturity and correlates with diminished gut barrier function in preterm infants, as assessed by sugar absorption tests [16]. In near term control tissue of 140d GA, staining of ZO-1 formed a continuous band lining the mucosal epithelium, indicating maturation of the gut barrier during gestation.

This study shows that bacterial toxins impair maturation of tight junctions during gestation, an effect which lasted as long as 30d post endotoxin administration. These data are in line with earlier in vitro and in vivo findings showing that the permeability of the intestinal epithelium is impaired after exposure to LPS [17]–[19]. An imperfect tight junctional distribution, either due to immaturity or inflammation suggests an easy access of microbial toxins to the mucosa and the inner layers of the gut, both in utero and after birth. This led us to investigate the mRNA levels of the molecules representing the endotoxin recognition complex during gestation and after intraamniotic endotoxin injection. A gestational dependent increase of intestinal TLR4 and MD-2 mRNA was detected. In line, recent findings from our group showed that the human MD-2 protein is expressed in term but absent in preterm infants [20]. Combined, these findings suggest that the capacity to recognize and sense Gram-negative derived endotoxin by the fetal intestine is immature at low GA. Interestingly, within 48 h after intraamniotic endotoxin, TLR4 mRNA became undetectable and concomitantly, also MD-2 expression levels were reduced. The nature of this rapid downregulation needs further investigation [21], [22], but it might explain the here observed hyporesponsiveness of the fetal gut to endotoxin at low GA. In particular, an impaired recruitment of MPO-positive cells or T lymphocytes was detected in the intestine at 2d after intraamniotic injection of endotoxin at 125d GA, likely due to absence of endotoxin recognition. This is consistent with earlier studies in this chorioamnionitis model showing that proinflammatory cytokine mRNAs were unaltered in the fetal intestine, from five hours to 25d after intraamniotic injection of endotoxin [23]. In contrast to the intestinal TLR4 and MD-2 mRNA expression data presented here, previous studies with this animal model showed that intraamniotic endotoxin induced pulmonary TLR4 mRNA expression within 2d [24]. In line, endotoxin induced chorioamnionitis provoked a rapid inflammatory reaction in the respiratory system within 24 hours [5], [6], [25], [26].

Interestingly, at 14 and even 30d after intraamniotic endotoxin injection, a strong intestinal inflammatory response was present with a massive influx of MPO positive cells and T-lymphocytes. Paradoxically, at 14 and 30d post endotoxin exposure, TLR4 mRNA was still undetectable while MD-2 expression levels remained reduced. Whether this sustained inflammatory process is the result of a broken or absent tolerance to endotoxin remains to be elucidated [22], [27]. In addition, activation via TLR4-MD-2 independent intestinal innate immune receptors cannot be excluded with these experiments. Alternatively, the delayed inflammatory response in the fetal gut might be the consequence of a systemic rather than a local response of the fetal intestine. Esophageal occlusion experiments need to be performed to define the site of stimulation of the massive influx of PMNs and T-cells in the fetal gut.

At 30d post endotoxin exposure, inflammatory parameters were decreased compared to the 14d group, and this was paralleled by migration of gammadelta T-cells from the lower mucosa towards compromised epithelial surfaces. These findings are supported by a report from Chen et al. who showed that gammadelta T-cells preserve the integrity of stressed or injured intestinal mucosa [12]. Moreover, our study showed that the lymphoid follicles of the healthy fetal intestine became populated with gammadelta T-lymphocytes between 125 days and 140 days of gestational age. In addition to the fragmented tight junctional distribution as seen in preterms or following endotoxin treatment, the absence of gamma delta T-cells in the mucosa at 125d GA suggests a reduced capacity to preserve the already compromised epithelium.

This study shows that intrauterine exposure to endotoxin induced a transient increase in the mesenteric vascular responsiveness to the thromboxane receptor agonist U46619. In addition a profound suppression of ACh-induced endothelium-dependent relaxation in mesenterial arteries was found during chorioamnionitis at low GA a process that was completely reversed by administration of the NO donor SNP. Our results strongly suggest the presence of an endotoxin-evoked transient endothelial damage and it could be speculated that a similar mechanism of endothelial dysfunction and diminished NO production might be partially responsible for the vascular vulnerability of the intestine in preterm infants [28].

The decreased NO availability could have several explanations. First, clinical reports showed that the intestinal synthesis of the important NO precursor arginine is strongly reduced in premature infants [29]–[32]. Second, a mechanism for the reduced NO biosynthesis would be a decreased expression of eNOS. Paradoxically, our data showed an enhanced expression of eNOS in the chorioamnionitis exposed animals. Such an increase in eNOS expression indicates the presence of a compensatory mechanism, which would attempt to maintain normal NO level by upregulating eNOS [33], [34].

In summary, in this study evidence is provided that both prematurity and chorioamnionitis are associated with impaired development of the intestinal innate immune defence, the tight junctional distribution and vascular function in utero. Although at present, we can only speculate about the postnatal consequences of these developmental disorders, immaturity of the parameters investigated in this study are all associated with neonatal intestinal pathology and NEC in particular [35]–[38]. The immature gut of preterm babies is a risk factor on its own which is further aggravated by the presence of antenatal inflammation. In addition, reduced expression of innate immune receptors as seen in the intestine of preterm lambs suggests that the capacity to recognize and sense Gram-negative derived endotoxin by the fetal intestine is immature at low GA. Such a lack of endotoxin sensing in the preterm intestine, against the background of a disrupted tight junctional distribution, supports the theory that an inadequate immune response contributes to the pathogenesis of NEC, potentially by allowing bacterial invasion and subsequent overgrowth in premature infants. Further research is needed to assess the importance of preterm delivery and the consequences of inflammation in utero on the development of neonatal intestinal pathologies such as NEC.
